# Draft Genome Sequences of *Halobacterium* sp. Strains KA-4 and KA-6, Two Extremely Halophilic Archaea Isolated from a Triassic Salt Deposit in Northern Ireland

**DOI:** 10.1128/mra.01165-21

**Published:** 2022-01-20

**Authors:** Fintan T. McMahon, Ciaran M. Lonergan, Brendan F. Gilmore, Julianne Megaw

**Affiliations:** a School of Biological Sciences, Queen’s University Belfast, Belfast, United Kingdom; b School of Pharmacy, Queen’s University Belfast, Belfast, United Kingdom; Portland State University

## Abstract

Here, we report the draft genome sequences of *Halobacterium* sp. strains KA-4 and KA-6. These extremely halophilic archaea were isolated from a Triassic halite deposit in Northern Ireland. Based on 16S sequence identity, they were deemed to be closely related strains of Halobacterium noricense but with some notable phenotypic differences.

## ANNOUNCEMENT

The genus *Halobacterium* contains extremely halophilic archaea; it was first described in 1957 and emended in 2009 (1). To date, the genus contains four species (2). Representatives of the genus have been isolated from diverse hypersaline environments, including halite deposits (3, 4), salt lakes (5), solar salterns (6), salted hides, and ancient parchments (7).

The extremely halophilic archaeal strains KA-4 and KA-6 were isolated from a brine sample taken from Kilroot Salt Mine, a Triassic halite deposit located in Northern Ireland, as previously described (4). Both strains had rod-shaped cells and red coloration, and both were oxidase negative and catalase positive (determined using oxidase detection strips [Oxoid, UK] and 3% H_2_O_2_, respectively). The strains were maintained on Payne’s medium (8), and both showed optimal growth when this medium contained 200 g L^−1^ NaCl (approximately 23% total salts), as determined by measurements of the optical density at 600 nm (OD_600_) following 7 days’ growth in this medium supplemented with 0, 5, 10, 15, 20, or 25% (wt/vol) NaCl. The strains were confirmed to be members of the genus *Halobacterium* based on a comparison of their 16S rRNA gene sequences to others held within the NCBI database (4). The closest neighbor to both strains was deemed to be the type strain Halobacterium noricense JCM 15102 (GenBank accession number NR_113426.1), with KA-4 having 99.49% sequence similarity and KA-6 having 99.56% similarity to this strain. The 16S sequences of both isolates also had 99.56% similarity to each other; however, considerable phenotypic differences were observed, most notably their antimicrobial susceptibility profiles toward novobiocin, rifampicin, and H_2_O_2_, measured using a standard MIC assay (9) (Table 1).

**TABLE 1:** Antimicrobial susceptibility data, sequencing and assembly metrics, and NCBI PGAP annotation data for *Halobacterium* sp. strains KA-4 and KA-6

Characteristic^*a*^	Data for strain:	
	KA-4	KA-6
MICs		
Novobiocin (ng mL^−1^)	0.24	0.12
Rifampicin (μg mL^−1^)	0.016	0.002
H_2_O_2_ (% [wt/vol])	0.032	0.002
		
Sequencing and assembly metrics		
Sequencing coverage (×)	84.0	73.7
Assembly size (bp)	3,336,915	3,492,034
No. of contigs (>1,000 bp)	55	46
Largest contig (bp)	387,766	871,498
*N*_50_ (bp)	138,062	362,719
*L*_50_	8	3
GC content (%)	63.8	63.9
		
NCBI PGAP annotation data		
No. of identified genes	3,598	3,745
No. of identified CDSs	3,546	3,694
No. of complete RNAs (5S, 16S, 23S)	1, 1, 1	1, 1, 1
No. of predicted tRNAs	47	46
No. of predicted ncRNAs	2	2

a CDSs, coding DNA sequences; ncRNAs, noncoding RNAs.

Both isolates were grown in Payne’s medium (200 g L^−1^ NaCl) at 37°C for 7 days, and genomic DNA was extracted using the GenElute bacterial genomic DNA kit (Merck, UK) as per the manufacturer’s instructions, using the protocol for Gram-negative bacteria. Whole-genome sequencing was performed by Microbes NG (Birmingham, UK). Genomic DNA libraries were prepared using the Nextera XT library prep kit (Illumina, San Diego, CA) and sequenced using the Illumina MiSeq/NovaSeq platform, with a 250-bp paired-end protocol. All tools for assembly and analysis were run using default parameters unless otherwise specified. The reads were adapter trimmed using Trimmomatic v0.30 (10) with a sliding window cutoff of Q15. The reads were assembled *de novo* using SPAdes v3.7 (11), and contigs of <1,000 bp were discarded. The two assemblies were then annotated using the NCBI Prokaryotic Genome Annotation Pipeline (PGAP) v5.3 (12). The sequencing and assembly metrics and key features of the two genome assemblies are summarized in Table 1. These two draft genomes will contribute to the increasing number and diversity of understudied haloarchaeal genomes currently available for analysis.

### Data availability.

The raw Illumina reads for KA-4 and KA-6 have been deposited in the Sequence Read Archive (SRA) under accession numbers SRR16917818 and SRR16917817, respectively. The draft genome sequences have been deposited at DDBJ/ENA/GenBank under accession numbers JAJJZG000000000 and JAJJZH000000000, respectively. The versions described in this paper are JAJJZG000000000.1 (KA-4) and JAJJZH000000000.1 (KA-6).
